# What is in your wallet? A cluster randomized trial of the effects of showing comparative patient out-of-pocket costs on primary care prescribing for uncomplicated hypertension

**DOI:** 10.1186/s13012-017-0701-x

**Published:** 2018-01-10

**Authors:** Robyn Tamblyn, Nancy Winslade, Christina J. Qian, Teresa Moraga, Allen Huang

**Affiliations:** 1 Division of Clinical Epidemiology, McGill University Health Centre, Montreal, QC Canada; 2 Department of Epidemiology, Biostatistics and Occupational Health, McGill University, Montreal, QC Canada; 3 Clinical and Health Informatics Research Group, McGill University, Montreal, QC Canada; 4 Division of Geriatric Medicine, McGill University, Montreal, QC Canada; 5 Division of Geriatric Medicine, University of Ottawa, Ottawa, ON Canada; 6 McGill University, Morrice House, 1140 Pine Ave West, Montreal, QC H3A 1A3 Canada

**Keywords:** Computerized decision support, Electronic prescribing, Cost-effectiveness, Anti-hypertensive agents

## Abstract

**Background:**

Drug expenditures are responsible for an increasing proportion of health costs, accounting for $1.1 trillion in annual expenditure worldwide. As hundreds of billions of dollars are being spent each year on overtreatment with prescribed medications that are either unnecessary or are in excess of lowest cost-effective therapy, programs are needed that optimize fiscally appropriate use. We evaluated whether providing physicians with information on the patient out-of-pocket payment consequences of prescribing decisions that were in excess of lowest cost-effective therapy would alter prescribing decisions using the treatment of uncomplicated hypertension as an exemplar.

**Methods:**

A single-blind cluster randomized trial was conducted over a 60-month follow-up period in 76 primary care physicians in Quebec, Canada, and their patients with uncomplicated hypertension who were using the MOXXI integrated electronic health record for drug and health problem management. Physicians were randomized to an out-of-pocket expenditure module that provided alerts for comparative out-of-payment costs, thiazide diuretics as recommended first-line therapy, and tools to monitor blood pressure targets and medication compliance, or alternatively the basic MOXXI system. System software and prescription claims were used to analyze the impact of the intervention on treatment choice, adherence, and overall and out-of-pocket payment costs using generalized estimating equations.

**Results:**

Three thousand five-hundred ninety-two eligible patients with uncomplicated hypertension were enrolled, of whom 1261 (35.1%) were newly started (incident patient) on treatment during follow-up. There was a statistically significant increase in the prescription of diuretics in the newly treated intervention (26.6%) compared to control patients (19.8%) (RR 1.65, 95% CI 1.17 to 2.33). For patients already treated (prevalent patient), there was a statistically significant interaction between the intervention and patient age, with older patients being less likely to be switched to a diuretic. Among the incident patients, physicians with less than 15 years of experience were much more likely to prescribe a diuretic (OR 10.69; 95% CI 1.49 to 76.64) than physicians with 15 to 25 years (OR 0.67; 95%CI 0.25 to 1.78), or more than 25 years of experience (OR 1.80; 95% CI 1.23 to 2.65). There was no statistically significant effect of the intervention on adherence or out-of-pocket payment cost.

**Conclusions:**

The provision of comparative information on patient out-of-pocket payments for treatment of uncomplicated hypertension had a statistically significant impact on increasing the initiation of diuretics in incident patients and switching to diuretics in younger prevalent patients. The impact of interventions to improve the cost-effectiveness of prescribing may be enhanced by also targeting patients with tools to participate in treatment decision-making and by providing physicians with comparative out-of-pocket information on all evidence-based alternatives that would enhance clinical decision-making.

**Trial registration:**

ISRCTN96253624

## Background

Drug expenditures are responsible for an increasing proportion of health costs, accounting for $1.1 trillion in annual expenditure worldwide. Even with projected slowed growth from 9% in 2014 and 2015 to about 4–7% over the next 5 years, it will reach approximately $1.5 trillion by 2021 [1–3]. Health care systems in many countries face the same challenge—health system sustainability when cost increases outstrip economic growth [4]. Health systems need to provide adequate access to essential medication for their citizens in a fiscally responsible and sustainable manner. Programs are needed that can support optimal appropriate prescribing and discourage unwarranted drug expenditures. For example, in 2011, approximately $158 to $226 billion were spent in overtreatment in the USA alone [5–9].

Governments and third-party payers have led most of the initiatives to improve drug use and control drug costs [5, 7, 10, 11], primarily through consumer-directed user fees such as deductibles, co-payments, co-insurance, and/or spending caps [12–14]. User fees are intended to discourage the use of marginal therapies that have limited effectiveness in maintaining or improving health status [7, 12, 13, 15]. Systematic reviews of the effects of cost-sharing policies have shown that these interventions have led to reductions in medication adherence for both essential and less essential therapy [12, 13, 15–17], with a commensurate increase in medical visits, emergency care, and hospital admissions [13, 15–17]. For this reason, consumer-directed policies, such as cost-sharing, are seen to play a limited role in optimizing prescription drug use.

Physician-directed policies should be more successful in improving cost-effective use of medications since almost all prescribing decisions are made by physicians [15, 18]. Tiered formularies and reference-based pricing are two of the most commonly used policies that aim to influence the choice of medication prescribed towards more “cost-effective therapies” [19–23]. Both of these strategies use out-of-pocket payments by patients to influence physician treatment choices—where higher out-of-pocket payments are required for drugs that are considered to be less “cost-effective.” Several flaws with these popular approaches have been identified. Physician knowledge of the cost of drugs they prescribe is poor [24–27], even though most physicians consider cost to be an important factor in prescribing decisions [27, 28], particularly when it affects their patients’ out-of-pocket payments [29]. As a result, the pharmacists or third-party benefit managers then assume the responsibility for calling physicians to request changes in drugs prescribed to adhere to drug choice policies [25]. These call-backs to physicians are time and labor-intensive [25, 30, 31]. Patients frequently decide to lower their out-of-pocket expenses by discontinuing medication or to ration use of drugs with higher co-payments [21–23, 32–37]. These patient-directed solutions result in unintended consequences since the majority of medications are used for chronic disease management, and under-use of essential therapy is associated with an increased risk of avoidable morbidity [38–43].

Physician-directed cost containment could potentially be more effective if physicians knew at the time of prescribing their patients’ expected out-of-pocket expenditure [44, 45]. Computer-assisted prescribing and decision-support systems can be designed to display cost information at the time of prescribing, but there has been limited study of the potential value of this tactic in primary care where the majority of drugs are prescribed [45–48]. Of interest, although recommended [44, 45, 49], no intervention to date has specifically addressed one of the most important elements—physician’s knowledge of the consequences of prescribing decisions on the out-of-pocket costs for their patients. Observational studies suggest that physicians will prescribe lower-cost medication or use samples to reduce financial burden [29, 50], when it is the patient who has to pay rather than the insurance company [50]. However, it has been difficult to provide out-of-pocket payment information at the time of prescribing as factors involving the patient’s insurance plan, deductible, and co-payment requirements can be independently modified with little notice. With the recent implementation of electronic prescribing and integrated drug management systems at prototype centers in Quebec, it is now possible to develop and test the potential benefit of providing physicians with comparative out-of-pocket costs for patients at the time of prescribing.

We tested the hypothesis that the provision of comparative information on expected patient out-of-pocket expenditures to physicians at the time of prescribing will increase the proportion of patients started on or switched to more cost-effective treatment and improve medication adherence. The treatment of uncomplicated hypertension was selected as a test case because (1) multiple drugs exist to treat the same problem, (2) substantial differences exist in treatment costs among available choices, (3) a large proportion of the population is treated for the condition, (4) level-one evidence exists from randomized controlled trials showing that treatment among the available options is equivalent, and (5) a substantial proportion of the population are not receiving the most cost-effective therapy [51–56].

## Methods

### Context

This study was conducted in the province of Quebec, Canada. The provincial health insurance agency, Régie de l’assurance-maladie du Québec (RAMQ), provides coverage for all eligible residents and pays all physicians and community pharmacists on a fee-for-service basis. All provincial residents are required to have drug insurance, either through enrollment in their employer’s benefit plans, or if not available, in the RAMQ public plan. Approximately 50% of residents are enrolled in the public plan, including all persons 65 years of age or older, welfare recipients, and persons who could not be insured through their employer. In the public plan, welfare recipients and seniors who receive a full guaranteed income supplement from the government are not required to share in the cost of their prescriptions. All other beneficiaries are required to share in the cost of their prescriptions which, during the period of this study, comprised a fixed monthly deductible of $16.50 and a co-pay of 25% of the cost of the drug plus the pharmacist’s dispensing fee. If a beneficiary’s out-of-pocket costs in a given month exceeded $52.65 for those receiving a partial government income supplement or $88.83 for those receiving no income supplement, the full costs of all additional prescriptions would be covered by the RAMQ plan.

Data generated by administrative claims for pharmacist and physician reimbursement in the RAMQ health and drug insurance program have been validated for clinical and research use [57–61]. In 2003, an experimental primary care-based electronic prescribing solution, the Medical Office of the XXIst century (MOXXI), was established and was the first system to connect to administrative health databases and integrate this information into a clinical electronic health record system [62].

### Design and study population

A single-blind, cluster randomized controlled trial was conducted over a 60-month follow-up period to test the hypothesized benefits of providing comparative patient out-of-pocket expenditure information at the time of prescribing anti-hypertensive medication. Both newly treated (incident), defined as those patients having no anti-hypertensive prescriptions or dispensed medications in the prior 12-months, and currently treated (prevalent) patients, those with evidence for active therapy in the prior 12-months, were studied. The trial was conducted within an existing cohort of primary care physicians and their patients in the two urban centers of Montreal and Quebec City. We based our sample size on the ability to detect a minimum difference of 10% (an increase from 20 to 30%) in the proportion of patients with uncomplicated hypertension who are prescribed thiazides for hypertension management.

Physicians were eligible for inclusion if they were participants in the MOXXI electronic health record platform. This cohort, comprised of 76 full-time community-based primary care physicians in fee-for-service practice, represents 25% of all eligible primary care physicians within the geographically defined areas of the two cities. Patient inclusion criteria included age 18 years or older, insured by RAMQ and were required to share the cost of their prescriptions, made one or more visits to an enrolled study physician during follow-up, had a diagnosis of uncomplicated hypertension in the year before or at the visit (i.e., hypertension recorded by the study physician in the therapeutic indication field that must be completed with each prescription), and the study physician was the prescriber of their anti-hypertensive therapy. Patients were excluded if the following comorbidities were identified at the time of enrollment, as treatment regimens differ for complicated hypertension: diabetes, congestive heart failure, atherosclerotic disease, peripheral arterial disease, ischemic heart disease—angina or prior myocardial infarction, past cerebrovascular accident or transient ischemic attack, renal disease, asthma, chronic obstructive pulmonary disease (COPD), or left ventricular hypertrophy. Patient eligibility was determined at each visit during follow-up by an automated review of changes to the electronic problem list [61, 63]. Once a patient was considered eligible, they were followed until the completion of the study, death, admission to long-term care, or a move out of the province, whichever came first. All costs are reported in Canadian dollars.

### Intervention and control group

#### Control group

The control group physicians had access to the basic computerized MOXXI medical record system but not the out-of-pocket expenditure module. MOXXI is a secure web-based electronic prescribing solution that provides a number of clinically useful features that increase the adoption and use of this technology in primary practice [63–66]. Information for each patient is retrieved in real-time from the RAMQ administrative databases, including their drug insurance coverage, dispensed prescriptions, and medical service claims. MOXXI presents drug information as a dynamic graphical display showing all currently active medications, color-coded by prescribing physician, as well as drug costs and dates of ER and inpatient hospital episodes based on medical service claims. A potential list of medical conditions and problems is generated from recorded treatment indications for drugs prescribed as well as by inclusion of ICD9 codes from medical service claims. This list is presented to physicians for validation. Prescription printouts are given to the patient and kept in the chart. To enhance efficiency, each patient’s electronic record is pre-populated with his or her demographic, drug, and medical visit information from the RAMQ claim data, and prescription refills are expedited by providing a “quick click” re-prescribing function for multiple repeat prescriptions. A commercial drug knowledge and alert system is integrated into the MOXXI system that provides physicians with drug monographs and medication review and alerts for potential drug interactions, therapy duplications, drug disease and allergy contraindications, and excessive doses. Alerts are classified into three levels of severity. The MOXXI system allows physicians to set the level of alerts they wish to see and eliminate alerts that they think are irrelevant for some or all patients. Most MOXXI physicians elect to see only the most severe alerts (absolutely contraindicated) that represent approximately 5% of all alerts [67].

#### Intervention group

A comparative *out-of-pocket expenditure module* was developed and incorporated into the MOXXI system to provide decision support to the prescribing physician (1) for selecting the most cost-effective drug for new anti-hypertensive treatment and (2) to facilitate switching patients who are currently treated for uncomplicated hypertension to more cost-effective therapy.

**Fig. 1:**
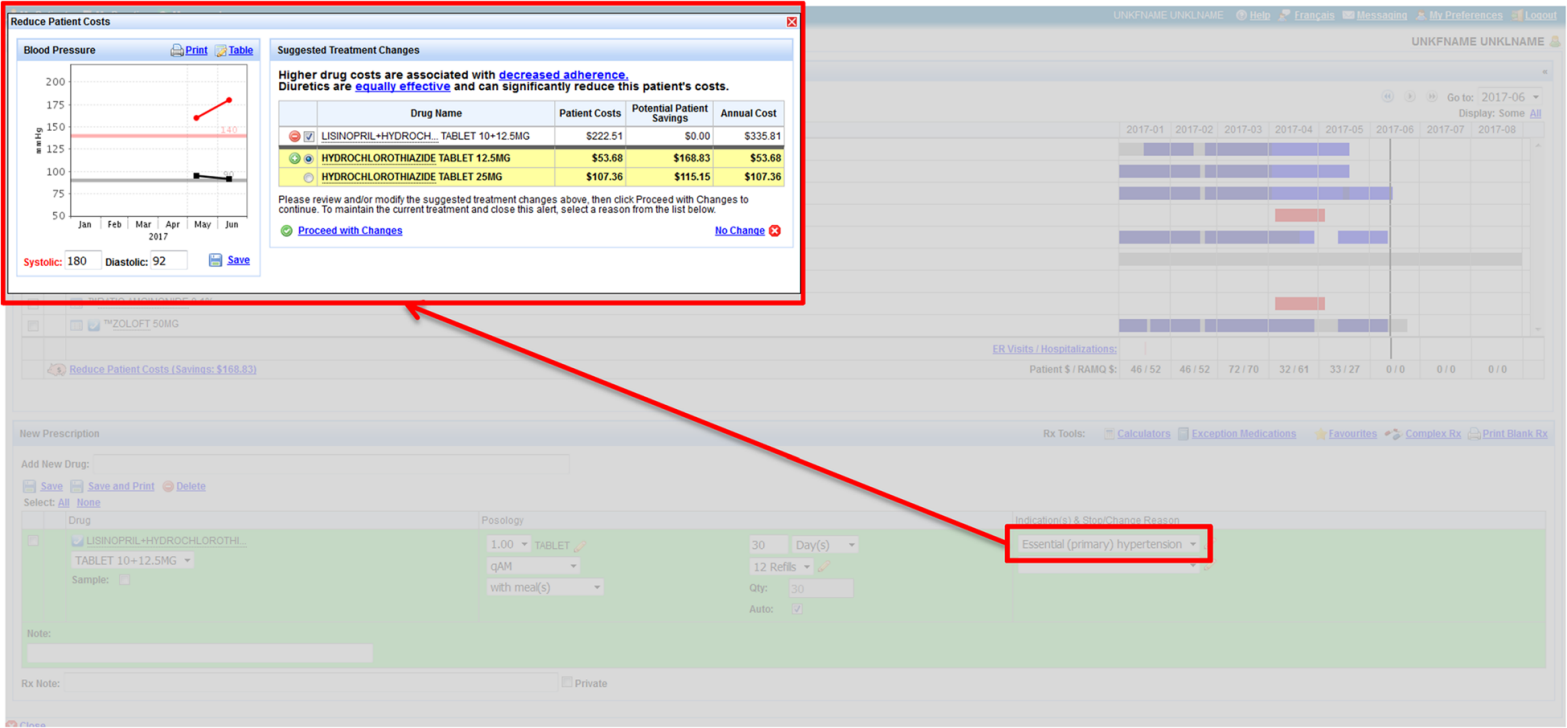
Decision support for newly treated hypertension patients. Pop-up with patient cost information upon selection of the indication “Essential (primary) hypertension”

**Fig. 2:**
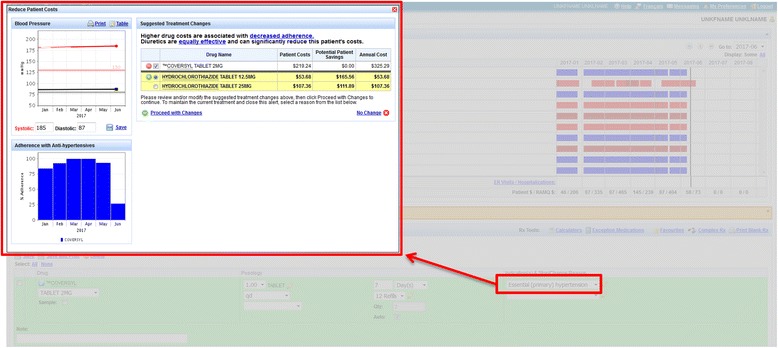
Decision support for currently treated hypertension patients. Pop-up with patient cost information upon selection of the indication “Essential (primary) hypertension”. Past adherence information is also available to allow a more informed decision

i) Decision support for newly treated hypertension patients: Decision-support recommendations were integrated into the electronic prescription pad. During typical use, a drop-down menu of drugs with their corresponding commonly prescribed dosages and frequencies appears as the physicians enter the first letters of the drug name. Once the physician finds and selects the desired drug sentence, the information is used to automatically populate the electronic prescription, which can be further modified if needed. If a physician is intending to prescribe a new anti-hypertensive agent for uncomplicated hypertension, a pop-up window will open automatically, showing alternate drug choices and the corresponding out-of-pocket and total costs of each treatment. Expected annual out-of-pocket costs are calculated based on the amount that the RAMQ will pay for the drug(s) (per tablet or capsule), the frequency selected, and the pharmacist’s dispensing fee. We assume the pharmacist will adhere to the recommended provincial practice of providing a 1-month supply (12 dispensings/year) [68]. We then apply the monthly deductible and 25% co-payment to the annual cost of the drug and pharmacist’s fees to determine annual out-of-pocket costs. The physician then has the option of selecting an alternative therapy and the system will automatically generate an electronic prescription for that selection (Fig. 1). The design of the intervention is based on prior findings that indicate that physicians want to be informed of more cost-effective option at the time of prescribing [25, 29] and are more likely to prescribe the more cost-effective option if the alternative option can be selected easily and quickly [69]. If physicians decided not to switch to a diuretic as first-line therapy, they were asked to document the reason for their decision.

ii) Decision support for currently treated hypertension patients: We anticipated that physicians are more reluctant to switch existing treatment for patients who have already been started on a less cost-effective therapy, unless the patient is not achieving the desired treatment response, is not adherent to treatment (possibly because of cost), and/or has difficulty paying the out-of-pocket costs. A pop-up window appears when the anti-hypertensive is being renewed. This window provides information about the patient’s current annual out-of-pocket payment and the annual savings that would occur with a change in treatment (Fig. 2). Out-of-pocket costs were calculated using the same method outlined for newly treated patients. Physicians can click on the highlighted medication to view medication compliance within the last 6 months, drug details and cost, and can click a button to switch patients to the recommended therapy. The resulting changes in treatment are displayed in the prescription preview and printed as part of the prescription transmitted to the pharmacy. If physicians decided not to switch treatment, they were asked to document the reason for their decision and the alert would then be suppressed.

### Randomization and blinding

Physicians were stratified by city, practice population size, and proportion of prevalent patients on diuretic treatment, with groupings sufficient to maintain a minimum of two physicians within each stratum. Within each stratum, we assigned a random number to each physician, and an equivalent number of physicians within each stratum were allocated to the intervention and control groups. Randomization took place 2 months prior to the start of the intervention, to permit sufficient time for physician training, and was carried out by an independent statistician, blinded to physician identity. Due to the nature of the intervention, it was not possible to blind either physicians or patients, but both were blinded to the primary and secondary outcomes of the study, as were the research assistants involved in training, implementation, and analysis, although physicians may have suspected that the primary outcome was an increase in diuretic use.

### Physician training and support

All physicians enrolled in the MOXXI program receive one-on-one training in their clinic on how to use MOXXI. Their practice population information is pre-loaded prior to the start of training. The effectiveness of training is determined by the completion of a standardized set of tasks in prescribing, stopping, and modifying drug treatment, and adding problems to the problem list, at the end of the training and 1 month later. A local technical and training team is on call to address problems. The team proactively monitors utilization rates and intervenes if utilization is low. The training team provided one-on-one training on how to use the out-of-pocket training module in the clinic, monitored its use, and periodically visited each practice to provide proactive support.

### Outcomes

#### Primary outcome: prescription of cost-effective treatment for uncomplicated hypertension

The intended effect of the intervention was to influence the physician’s choice of the drug prescribed to manage hypertension. We classified an individual’s anti-hypertensive drug treatment into one of three mutually exclusive categories: (1) thiazides alone, (2) other evidence-based but less cost-effective single drug therapy (angiotensin-converting enzyme inhibitors, beta blockers, calcium channel blockers, angiotensin 2 receptor blockers), and (3) other non-evidence-based choices including concurrent multiple anti-hypertensive drugs. These categories were derived from the 2016 Canadian Hypertension Education Program Clinical Guidelines [70]. Patients were classified according to drugs that were prescribed on the first visit during the follow-up period based on data retrieved from the MOXXI system. Drugs included in each category were defined by generic form and nationally standardized drug identification numbers and were updated to accommodate new medication in the therapeutic class during the study period.

#### Secondary outcome: adherence with prescribed treatment for uncomplicated hypertension

Adherence to drug treatment is adversely affected by higher out-of-pocket expenditures [16, 38–41, 55]. If the intervention produces meaningful reductions in the out-of-pocket expenditures for patients, then one would expect a corresponding improvement in treatment adherence. *Treatment adherence* was defined as proportion of days in which the patient had the expected supply of medication in the year following the visit in which the prescription was received [71, 72]. Records of prescribed, stopped, and dispensed drugs for each patient for the year following the first visit post-randomization and the 6 months prior to randomization were retrieved and used to create a drug by day matrix of expected and actual drug supply days as a function of prescribed therapy. When a patient was prescribed more than one drug in a therapeutic class, the mean adherence value was calculated for all drugs prescribed.

### Patient characteristics

Since physicians rather than patients were randomized, systematic differences may exist between intervention and control patients that may influence the outcome, as the characteristics of the practice population differ among physicians. To adjust for these potential differences between physician practice populations, the following patient characteristics were collected: age, sex, estimated household income, drug insurance plan, current treatment costs (for prevalent patients), comorbidities, and health care use in the year before their first visit to the physicians after randomization. Patient age and sex were obtained from the RAMQ beneficiary data. Capacity to pay for prescriptions is an important determinant of physician’s choice of medication [29] as well as the likelihood of patient adherence to drug therapy [21–23, 33–36]. Capacity to pay was determined by estimating the average household income in the six-digit postal code area of the patient’s residence (approximately 36 households) based on Canadian census data [30]. Baseline anti-hypertensive treatment costs for prevalent patients were measured as the average annual costs of anti-hypertensive drugs dispensed in the 12 months prior to randomization. Comorbidity was assessed using the Charlson comorbidity index [61, 73, 74], based on diagnostic codes recorded in medical service claims and hospitalization discharge records in the 12 months prior to the patient’s first visit post-randomization. Baseline health care service use included the number of ER visits, number of hospitalizations, number of prescribed medications, and continuity of care with the primary care physician in the 12 months prior to the first visit post randomization for each patient using previously published algorithms [75].

### Analysis

Descriptive statistics were used to evaluate differences in the baseline characteristics of participating physicians and patients in the two arms of the trial. Study hypotheses were tested using an intention-to-treat analysis, whereby all consenting patients who made at least one visit to the study physician during the follow-up period were included in the analysis. For all patients, longitudinal information on health care utilization and mortality was available through linked provincial health administrative databases [76].

Logistic and generalized linear regression models were respectively used to analyze the effect of the intervention on the primary (diuretic prescribed) and secondary (treatment adherence) outcomes. To account for clustering of patients within physician, multivariate models were estimated within a generalized estimating equation (GEE) framework [77, 78], assuming an exchangeable correlation structure. Newly treated and currently treated hypertension patients were analyzed separately in relationship to the primary and secondary outcomes. The treatment effect (intervention vs. control) was represented as a binary level variable and was included in GEE models along with adjustment for baseline differences in patient demographics, comorbidity, household income, number of medications taken, and copayment plan, as well as physician-level differences in average costs of prescribed anti-hypertensives, years in practice, sex, practice volume, and propensity to prescribe diuretics. We suspected that both the patient-level differences as well as physician-level differences would affect physicians’ willingness to influence out-of-pocket costs [79–81]. In addition, to determine if the effect of the intervention was modified by these characteristics, we fit two-way interaction terms between each patient and physician characteristic with the intervention and assessed significance based on the model fit as well as the interaction term estimates.

## Results

**Fig. 3:**
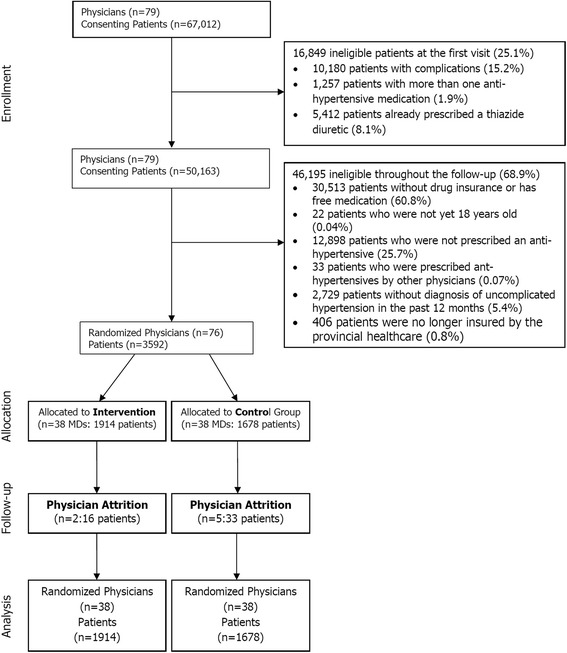
Physicians and patients eligible and enrolled in the trial: February 2009 to July 2015

Seventy-nine physicians were enrolled in this study. 25.1% of their 67,012 patients with hypertension were excluded on the first visit because they had complicated hypertension, were already using a diuretic, or were prescribed multiple anti-hypertensive therapies (Fig. 3). Of the remaining 50,163 patients, 60.8% either were not covered by the public drug plan or were receiving free medication. Of the remaining patients, 3592 had a diagnosis of uncomplicated hypertension and were prescribed anti-hypertensive treatment during the follow-up period by the study physicians. Seven physicians retired during the follow-up period and their data were included in the analysis.

Study physicians in the intervention and control groups were similar in sex, language, number of years in practice, practice size, and daily patient visits, as well as the estimated household income of their patients (Table 1). The mean number of patients being treated for hypertension at the start of the study was 152.7 in the control and 187.6 in the intervention group. For these patients, the most commonly prescribed anti-hypertensive treatment was calcium channel blockers (control group 21.7%, intervention 19.1%) followed by diuretics and beta blockers. Mean annual costs of anti-hypertensive treatment were $370.90 in the control group and $385.70 in the intervention group. Annual out-of-pocket patient costs were similar.

**Table 1:** Characteristics of the 76 physicians in the intervention and control groups, as well as the baseline characteristics of all patients under their care

	Control *N* = 38		Intervention *N* = 38	
Demographics	*N*	%	*N*	%
Sex				
Female	17	44.7	14	36.8
Male	21	55.3	24	63.2
Language				
English	11	28.9	9	23.7
French	27	71.1	29	76.3
	Mean	SD	Mean	SD
Years in practice	24.9	8.1	27.5	8.3
Practice characteristics	Mean	SD	Mean	SD
Annual practice size	1245.4	581.6	1328.8	719.9
Number of patients with hypertension^b^	152.7	115.2	187.6	145.2
Number of patients/clinic day	14.9	6.1	15.3	5.9
Mean household income (CAD$)	52,961	13,582	53,308	14,084
All patients with hypertension^b^	Mean	SD	Mean	SD
Mean proportion by therapeutic class				
Calcium channel blockers	21.7	10.8	19.1	9.1
Diuretics	19.9	10.1	15.6	9.9
Beta blockers	15.2	7.4	14.1	7.3
ACE inhibitors	14.3	8.3	11.9	7.3
Angiotensin II antagonists	12.4	6.9	13.8	7.6
Angiotensin II antagonist + diuretic	12.1	8.7	14.7	7.7
Angiotensin-converting enzyme	4.3	3.4	3.6	3.3
Inhibitor + diuretic	2.9	1.8	2.1	2.4
Other^a^				
Annual cost anti-hypertensive treatment				
Total annual cost (CAD $)	532.2	423.4	546.4	428.3
Annual co-payment cost (CAD $)	370.9	317.6	385.7	321.8

^a^The “Other” category includes alpha-2 agonist, beta blockers + diuretic, alpha-1 antagonist, renin inhibitor, and vasodilator

^b^Includes all patients with hypertension, both complicated and uncomplicated, who were treated by the study physicians

Most eligible patients with uncomplicated hypertension were between 60 to 75 years of age, female, in the middle-income bracket, and with few comorbidities that would increase the risk of mortality (Charlson index value of 0) (Table 2). Continuity of care was moderate, with 38.6% (intervention) and 41.1% (control) of all visits being made to the primary care physician, who was responsible for only 49.8% (control) to 50.0% (intervention) of all drugs prescribed. The proportion of patients with one or more ED visits and/or hospitalizations was similar in the intervention and control groups. Between 32.7% (intervention) and 37.9% (control) of patients were started on therapy in the follow-up (incident patients) period. For patients already prescribed anti-hypertensive medication (prevalent patients) at the start of the study, a mean of 1.3 different anti-hypertensives was prescribed concurrently; 94.3% (intervention) and 93.6% (control) of which were prescribed by the patient’s study physician.

**Table 2:** Baseline characteristics of the 3592 hypertension patients in the intervention and control groups between 2009 and 2015

	Control *N* = 1678		Intervention *N* = 1914	
Demographics	*N*	%	*N*	%
Age				
< 60 years old	449	26.8	474	24.8
60 to 74 years old	870	51.8	1044	54.5
> 74 years old	359	21.4	396	20.7
Sex				
Female	891	53.1	1100	57.5
Male	787	46.9	814	42.5
Language				
English	223	13.3	115	6.0
French	1455	86.7	1799	94.0
Estimated family income				
Low income (< $35,000)	344	20.5	457	23.9
Middle income ($35,000–$80,000)	1148	68.4	1272	66.5
High income (> $80,000)	186	11.1	185	9.7
Comorbidity				
Charlson index value				
0	1121	66.8	1255	65.6
1	388	23.1	443	23.1
1+	169	10.1	216	11.3
Diabetes	292	17.4	341	17.8
COPD	119	7.1	153	7.9
Any tumor (except metastatic)	101	6.0	133	6.9
Metastatic solid tumor	41	2.4	30	1.6
Connective tissue disease	18	1.1	25	1.3
Peripheral vascular disease	13	0.8	17	0.8
Dementia	9	0.5	12	0.6
Leukemia/lymphoma	12	0.7	11	0.6
Mild liver disease	5	0.3	8	0.4
Ulcer disease	2	0.1	4	0.2
Renal disease	–	–	1	0.1
HIV positive	2	0.1	2	0.1
Diabetes with end organ damage	1	0.1	4	0.2
Hemiplegia	1	0.1	–	–
Health care use in the year prior to first eligible visit	Mean	SD	Mean	SD
Medical visits				
Total number of visits	6.3	6.5	6.3	6.7
Mean % to study physician	41.1	34.4	38.6	33.6
Number of distinct physicians seen	3.4	3.3	3.6	3.3
Prescriptions				
No. of medications prescribed	2.3	3.4	1.4	2.7
No. of medications dispensed	4.6	4.7	4.7	4.6
Percentage of dispensed prescribed by study physician	49.8	45.2	50.0	44.9
Hospitalization(s)	*N*	%	*N*	%
Yes	181	10.8	196	10.2
No	1497	89.2	1718	89.8
ER visit(s)				
Yes	375	22.3	407	21.3
No	1303	77.7	1507	78.7
Status of anti-hypertensive drug use at first visit (ICD9 code = 401)				
Incident^a^	636	37.9	625	32.7
Prevalent^b^	1042	62.1	1289	67.3
Hypertension management 12 ms before the first visit (prevalent)	Mean	SD	Mean	SD
No. of different anti-hypertensives prescribed	1.3	0.6	1.3	0.5
No. of different anti-hypertensives dispensed	1.3	0.5	1.3	0.5
Percentage of different dispensed anti-hypertensives prescribed by study physician	93.6	19.1	94.3	17.9

^a^The patient does not have a dispensed anti-hypertensive over the past 6 months and has a diagnosis of essential hypertension in their problem list (ICD9 = 401)

^b^The patient has dispensed anti-HNT over the past 6 months and has a diagnosis of essential hypertension in their problem list (ICD9 = 401)

**Fig. 4:**
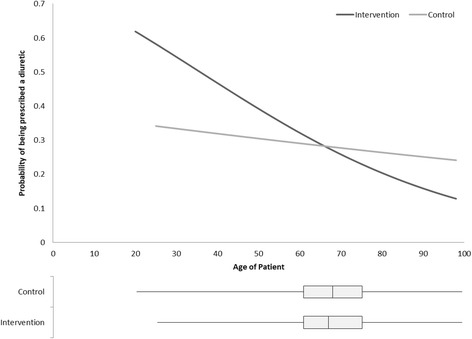
Probability of being prescribed a diuretic among currently treated intervention and control patients as modified by the age of patients. Covariates adjusted include gender, age of patient, Charlson comorbidity index, household income, number of medications, co-payment amount (maximum vs. partial/minimum), cost of total anti-hypertensive medications prescribed at the physician’s practice, physician’s level of experience, and physician’s propensity to prescribe diuretics, as well as the interaction term between the age of patient and the intervention

Among newly treated patients, 26.6% were prescribed a diuretic in the intervention group compared to 19.8% in the control group, a significant increase of 65% (adjusted OR 1.65, *p* = 0.01) in the odds of prescribing diuretics in the intervention group after adjusting for patient and physician characteristics (Table 3). Among patients who had already been started on anti-hypertensive therapy before the start of the study, 15.5% (intervention) and 15.3% (control) were switched to diuretics, a non-statistically significant change in the odds of diuretic prescribing (OR 1.09, *p* = 0.60). Incident and prevalent patients in the intervention group were also more likely to be prescribed a single anti-hypertensive compared to the control for both newly treated (intervention 85.4%; control 84.0%) and currently treated patients (intervention 77.4%, control 74.5%), although this difference was not statistically significant. We assessed whether the intervention was modified by age, comorbidity, level of co-payment required by the drug insurance plan, estimated household income, and the patient’s co-payment plan. There was a significant modification of the effect of the intervention by patient age among the prevalent patients. Older patients who had already been started on anti-hypertensive therapy were less likely to have their anti-hypertensive medication switched to a diuretic than patients under the age of 65 (Fig. 4). At the physician level, years of experience, sex, practice volume, average costs of prescribed anti-hypertensives, and prior rates of diuretic prescribing were assessed. Only the number of years in practice modified the effect of the intervention for newly treated incident patients. Physicians in practice less than 15 years were much more likely to prescribe a diuretic (OR 10.69; 95% CI 1.49 to 76.64) than physicians in practice 15 to 25 years (OR 0.67; 95% CI 0.25 to 1.78) or in practice more than 25 years (OR 1.80; 95% CI 1.23 to 2.65).

**Table 3:** Outcome estimates of the 3592 study patients by usage status at the end of 60-month observations

Outcomes	Incident, newly treated patients				
	Control (*n* = 636)	Intervention (*n* = 625)	Effect of intervention vs. control		
	*N* (%)	*N* (%)	Adjusted^a^ risk ratio	(95% CI)	*p* value
Therapeutic class					
Diuretic (at least one)	126 (19.8%)	166 (26.6%)	1.65	(1.17, 2.33)	0.004
Only other-hypertensive(s)	510 (80.2%)	459 (73.4%)	0.61	(0.43, 0.86)	0.004
No. anti-hypertensives					
I drug prescribed	534 (84.0%)	532 (85.1%)	1.10	(0.73, 1.66)	0.64
≥ 2 drugs prescribed	102 (16.0%)	93 (14.9%)	0.91	(0.60, 1.37)	0.64
	Mean (SD)	Mean (SD)	Adjusted^a^ risk diff	(95% CI)	*p* value
Treatment adherence					
Average adherence (%)	52.8 (34.7)	57.7 (34.1)	1.72	(− 6.91, 10.36)	0.70
Annual cost of therapy					
Total cost (CAD$)	441.6 (129.0)	453.4 (131.8)	0.78	(− 17.93, 19.49)	0.93
Out-of-pocket cost (CAD$)	252.6 (87.5)	261.5 (88.2)	0.27	(− 13.37, 13.91)	0.97
	Prevalent, currently treated patients				
	Control (*n* = 1042)	Intervention (*n* = 1289)	Effect of intervention vs.. control		
	*N* (%)	*N* (%)	Adjusted^a^ risk ratio	(95% CI)	*p* value
Therapeutic class					
Diuretic (at least one)	162 (15.3%)	200 (15.5%)	1.09	(0.79, 1.52)	0.60
Only other-hypertensive(s)	880 (84.7%)	1089 (84.5%)	0.91	(0.66, 1.27)	0.60
No. anti-hypertensives					
I drug prescribed	776 (74.5%)	998 (77.4%)	1.13	(0.90, 1.42)	0.28
≥ 2 drugs prescribed	266 (25.5%)	291 (22.6%)	0.88	(0.70, 1.11)	0.28
	Mean (SD)	Mean (SD)	Adjusted^a^ risk diff	(95% CI)	*p* value
Treatment adherence					
Average adherence (%)	70.7 (28.7)	72.1 (28.0)	1.36	(− 1.55, 4.28)	0.36
Annual cost of therapy					
Total cost (CAD$)	402.1 (149.7)	405.5 (160.0)	− 2.62	(− 19.59, 14.34)	0.76
Out-of-pocket cost (CAD$)	227.1 (98.4)	229.7 (103.4)	− 1.65	(− 12.59, 9.29)	0.77

Total of 3592 patients were included for these analyses, 1261 of which were incident, or newly treated patients, and 2331 were prevalent or currently treated patients

^1^Adjusted for age, gender, cci, household income, number of medications at baseline, copayment plan (maximum or partial/none), average annual practice anti-hypertensive cost, physician experience, and physicians’ propensity to prescribe diuretics

In relationship to secondary outcomes, mean adherence in the year after the first visit was higher in the intervention group for both incident (intervention 57.7%; control 52.8%) and prevalent patients (intervention 72.1%; control 70.7%); however, the difference in adherence after adjusting for patient and physician characteristics was not statistically significant (Table 3). There was also no statistically significant difference in the overall annual costs or out-of-pocket costs for anti-hypertensive therapy in the intervention and control groups for either incident or prevalent patients.

## Discussion

It has been postulated that the provision of information to physicians about individual patient’s out-of-pocket medication costs would influence their prescribing decisions. This study was the first to test this hypothesis using a sophisticated e-prescribing system that incorporated information on a patient’s concurrent medication, their drug insurance plan coverage, adherence to current medications, and alternate evidence-based options to therapy. Our target was the treatment, both incident and prevalent, of uncomplicated hypertension in community practice. We showed a significant increase in the prescribing of diuretics, the lowest out-of-pocket option for uncomplicated hypertension, for newly diagnosed patients. Among patients who were already on treatment, there was a significant effect on switching younger but not older patients to diuretics from other anti-hypertensive agents. The effect of the intervention was significantly greater for younger physicians and those in practice less than 15 years. They were more likely to prescribe diuretics for newly treated patients compared to similar physicians in the control group. There was no significant effect of the intervention on treatment adherence, total annual anti-hypertensive drug costs, or out-of-pocket payments. Further inspection of medications dispensed to newly treated patients in the 12-month follow-up period revealed that, compared with the control group, a higher proportion of patients in the intervention group had medications from other classes of hypertensives added to their initial prescription of diuretics. This might explain the observed lack of an effect of the intervention on total annual anti-hypertensive drug costs or out-of-pocket payments despite its effect on diuretic prescribing.

We evaluated the impact of providing comparative out-of-pocket cost information to physicians on their selection of treatment using uncomplicated hypertension as a case exemplar. Treatment of hypertension may not have been the best model as there is rapidly evolving evidence and controversy about guidelines for hypertension management, particularly in treatment targets and first-line treatment choices for uncomplicated and complicated hypertension [70, 82]. Hypertension treatment guidelines have changed over time. Initially, diuretics or beta blockers were recommended as first-line treatment of uncomplicated hypertension, and subsequently, calcium channel blockers, angiotensin-converting enzyme inhibitors, or angiotensin 2 receptor blockers were added as first-line treatment [70]. Hypertension treatment guidelines are susceptible to stakeholder interests [83], and the level of evidence for many recommendations does not meet grade A criteria [84]. Of interest, the effect of the intervention was significantly greater for younger physicians who are more likely to adopt electronic health record systems and computerized decision-support recommendations [85, 86], whereas older physicians are more likely to rely on industry-sponsored events for continuing professional development [87]. An early qualitative evaluation of hypertension management decision-making in a subset of this cohort of physicians provided some interesting insights about the factors that influence physician treatment decisions in hypertension management. Physicians who were less likely to prescribe diuretics believed these drugs were less efficacious, had more side effects, preferred treatment that would rapidly achieve their therapeutic target, and relied more on their own experience than the scientific evidence [83]. For these physicians, educational interventions may have limited success in altering prescribing behavior, which may explain the high failure rate in interventions to improve the cost-effectiveness of anti-hypertensive treatment [44, 45, 88]. An alternate approach would be to provide patients with the same information about the costs and out-of-pocket payments for alternative equally effective therapy. In comparison to physician-targeted interventions, this approach has been very successful in reducing the use of benzodiazepines in older adults [89]. Patient-centered approaches, particularly in the case of individual financial burden, should be considered in future interventions to improve cost-effective therapy.

This study was to provide physicians with salient information about out-of-pocket costs and the alternate evidence-based lowest cost alternatives at the time of making treatment decisions (incident patients), or when opening up the chart for the visit (prevalent patients). For incident patients, the computerized decision support was interruptive; the screen concerning alternate treatment and differences in cost would open without physician action (Fig. 1). These forms of alerts have been shown to be more effective than non-interruptive alerts [45, 90]. The significant interaction between the age of the patient and the effect of the intervention among these prevalent patients suggests that the reluctance to switch current treatment for older patients may be due to the fact that they may have already failed a trial of diuretics or the patients declined the treatment change. Informally, physicians in the intervention arm indicated that they would have preferred to have drug cost and out-of-pocket information for more than one evidence-based option, allowing them to use their clinical judgment in determining what would be best for an individual patient. We might have modified the intervention to address this concern had we done more user testing prior to implementation. Other studies have shown that synthesizing information to enable physicians to more easily visualize the risks and benefits associated with different treatment options has been successfully used in altering the prescription of psychotropic drugs in older adults [91]. More extensive user testing and the provision of information on all out-of-pocket costs for alternate choices should be considered in future computerized decision-support interventions to reduce unnecessary drug costs.

There are several limitations that need to be considered. The physician cohort was a non-random sample of primary care physicians practicing in the province of Quebec. While this will not bias the internal validity of the study, the results may not be generalizable to other jurisdictions. Our eligibility criteria for enrolling patients was strict, limiting the diagnosis to uncomplicated hypertension, absence of other comorbidities that may alter treatment choices and receiving treatment only from the primary care physician involved in the study. Many patients who started on hypertensive treatment in the study physicians’ practices were excluded because the single criterion of a diagnosis of uncomplicated hypertension was missing. Since these diagnoses were derived from the codes submitted along with the medical services claims and these codes have been shown to have high specificity and low sensitivity, more patients may have been eligible for inclusion into the study. Physicians were randomized, not clinics in order to obtain a better balance in physician and patient characteristics between the intervention and control groups. While physicians who were co-located in the same clinic do not usually share patient management except for weekend call, it is plausible that an intervention physician may have discussed out-of-pocket costs with a control physician. If these discussions modified the behavior of control physicians, contamination will have reduced observed differences between the two groups.

## Conclusions

In conclusion, the provision to physicians of comparative information on out-of-pocket payments for the treatment of uncomplicated hypertension resulted in a significant but modest impact on increasing the prescription of diuretics as initial therapy in newly diagnosed patients and the switch to diuretics from other agents in younger patients already on treatment. The impact of interventions to improve the cost-effectiveness of prescribing may be enhanced by also targeting patients with tools to participate in treatment decision-making and by providing physicians with comparative out-of-pocket information on all evidence-based alternatives.
